# Glycemic profile in patients with acromegaly treated with somatostatin analogue

**Published:** 2015

**Authors:** A Valea, M Carsote, C Ghervan, C Georgescu

**Affiliations:** * ”Iuliu Hatieganu” University of Medicine and Pharmacy; Clinical Country Hospital, Cluj-Napoca, Romania; ** ”Carol Davila” University of Medicine and Pharmacy, “C.I. Parhon” National Institute of Endocrinology, Bucharest, Romania

**Keywords:** acromegaly, octreotide, glycaemia, diabetes mellitus

## Abstract

**Hypothesis.** The growth hormone (GH) excess displayed in acromegaly induces insulin resistance up to diabetes mellitus (DM). The somatostatin analogues (as octreotide LAR) are useful in controlling the GH levels but disturbances of glucose metabolism might be seen.

**Objective.** This study evaluates the acromegalic glycemic profile under octreotide.

**Methods & Results.** Out of the total number of patients (N=34) diagnosed with active acromegaly, only some were followed (N=25; male/ female ratio: 6/ 19; mean age: 51.8 years) by testing GH, IGF1 (Insulin Growth Factor 1), basal glucose and oral glucose tolerance test (OCGTT) at baseline, 6 and 12 months under Octreotide (first 6 months with 20 mg/ 28 days + 6 months with 30 mg/ 28 days). Pre-treatment values were 17.6% of patients had DM, 14.7% - impaired glucose tolerance, 26.5% - impaired fasting glucose, and 41.2% - normal assays. From the statistical point of view, the DM patients were significantly older and had higher GH levels than the acromegalic without glycaemia disturbances. They did not achieve significant changes in basal blood glucose and glycated hemoglobin after 6 months, neither after 12 months.

After 6 months, there were no significant changes in basal glycaemia in patients with normal baseline glycaemia but 2-hours OGTT glucose values were significantly lower than initially (82.35 mg/ dl vs. 93 mg/ dl, p=0.005) consistent with reduced levels of GH and IGF1. After 12 months, both basal and 2-hours glucose levels in OGTT were similar to baseline despite the significant lower GH (3.3 vs. 6.61 ng/ mL, p=0.003) and IGF1 (332 vs. 713 ng/ mL, p=0.001).

**Conclusions.** Octreotide therapy induces an improvement in glycemic profile in patients with active acromegaly without diabetes mellitus consistent with decreased levels of GH and IGF1. In patients with diabetes, partial control of glucose metabolism is due to inadequate suppression of GH and IGF1 after one year of treatment.

## Introduction & Aim

Acromegaly is a classical endocrine disorder due to growth hormone excess seen after the growth cartilages are closed [**1**-**3**]. It is twice more frequent in women according to some studies and its prevalence is 5-7 cases in 100,000 people [**1**-**4**]. The condition associates metabolic disturbances in glucose and lipids metabolism increasing the cardiovascular risk [**1**]. In case of normal subjects, the growth hormone (GH) causes an increase of gluconeogenesis and lipolysis with consecutive high levels of glucose and free fatty acids, which further stimulates the insulin secretion despite augmented glycaemia [**2**]. Opposite to GH actions, the Insulin Growth Factor 1 (IGF1) increases insulin sensibility especially at muscle levels [**2**]. In acromegaly, the glucose tolerance is frequently altered; diabetes mellitus and impaired glucose tolerance are registered in 40-50% of the patients [**5**-**7**]. Persistent exposure to GH excess induces insulin resistance with secondary anomalies of hepatic and peripheral insulin activity [**8**]. The therapy in acromegaly is focused on lower GH levels and thus the insulin resistance and the associated cardio-metabolic risk [**9**,**10**]. After the first line option, which is pituitary surgery, the medical approach controls the GH and IGF1 levels in 60% of the cases [**9**,**10**]. The general data related to the precise effect on glucose metabolism of somatostatin analogues are still a matter of debate since in lower GH, we might expect a glycaemia improvement but by mimicking the physiological somatostatin, the pancreatic insulin is inhibited [**11**]. The study is aimed at observing the effect of the medical therapy in acromegalic glucose metabolism.

## Material & Method

This is an observational study in subjects admitted in a single tertiary endocrinology centre. The inclusion criteria were confirmation of acromegaly; therapy with somatostatin analogues: octreotide LAR in the first 6 months with 20 milligrams (mg) at every 28 days and then another 6 months with a single dose of 30 mg at every 28 days. The exclusion criteria were medical therapy of less than 1 year. The assays were blood GH (RadioImmunoAssay or RIA method), IGF1 (RIA method, age matched values, levels under 300 mg/ dL have been considered as normal), fasting glycaemia, and 75 grams (g) oral glucose tolerance test (OGTT) with both glucose and GH assays. All the measurements were performed at baseline (pre-treatment levels), and during follow-up after 6, respective 12 months of octreotide therapy. OGTT was not performed if the patient previously had diabetes mellitus, instead glycated haemoglobin (A1c) was measured and also 24-hours GH profile (a blood assay at every 4 hours). All the blood tests were performed with a day prior to the next octreotide injection. The American Diabetes Association (ADA) 2014 criteria were used to define diabetes mellitus (DM) as A1c ≥ 6.5% or fasting plasma glucose (FPG) ≥ 126 mg/ dL or 2-hour PG (plasma glucose) ≥ 200mg/ dL or a random PG ≥ 200 mg/ dL [**12**]. The categories between normal glucose profile and DM are pre- diabetes meaning impaired fasting glucose (IFG) with a FPG of 100 to 125 mg/ dL or impaired glucose tolerance (IGT) with a 2-h PG in 75-g OGTT of 140 to 199 mg/ dL [**12**]. Statistical analysis (Excel, SPSS) used student t-test with statistical significance at p<0.01. 

## Results

**Baseline analysis (pre-treatment)**

Out of 34 patients (male/ female ratio of 7/ 27) with active acromegaly, 25 were followed for 1 year (male/ female ratio of 6/ 19, mean age at baseline of 51.8 years. Applying ADA criteria 44% had normal glucose parameters; 28% had IFG, 16% had IGT and 12% DM (**Table 1**). 

**Table 1:** The glucose metabolism profile at baseline (N=25) in acromegalic patients (pre-treatment values)

Acromegaly	Normal Glucose Profile	IGT+IFG	DM
N=25	N=11 (44%)	N=4+7 (16%+28%)	N=3 (12%)
women N=19	N=9 (36%)	N=3+5 (12%+20%)	N=2 (8%)
men N=6	N=2 (8%)	N=1+2 (4%+8%)	N=1 (4%)
*IGT = Impaired Glucose Tolerance, IFG = Impaired Fasting Glucose, DM = Diabetes Mellitus *			

The mean age at diagnosis in patients with DM was 56 years versus 49 years in subjects with normal glycaemia (p<0.005). 3 groups were formed based on age at diagnosis: 30-50 years, 51-60 years, 61-70 years (most of subjects were in the first group) (**Table 2**).

A higher frequency of pre- diabetes was seen in 51-60 years group and DM was twice more frequent as normal glucose profile in the group with oldest subjects (**Table 2**).

**Table 2:** The glucose metabolism profile at baseline (N=25) in acromegalic patients (pre-treatment values)

Age groups (years)	Number of patients N=25	Normal glucose profile N=11	IGT+IFG N=4+7	DM N=3
30-50	14	8 (57%)	6 (42.8%)	1 (7%)
51-60	4	2(50%)	3 (75%)	0
61-70	7	1 (14%)	2 (28.6%)	2(28%)
*IGT = Impaired Glucose Tolerance, IFG = Impaired Fasting Glucose, DM = Diabetes Mellitus *				

**Follow-up analysis (at 6 and 12 months)**

**Fig. 1:**
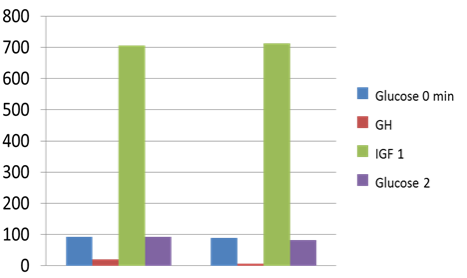
Fasting Glucose (Glucose 0 min; mg/ dL) and 2-hour Plasma Glucose (Glucose 2; mg/ dL) in 75 g OGTT; GH (ng/ mL) and IGF1 (ng/ mL) levels after 6 months of octreotide LAR (a dose of 20 mg at every 28 days) in patients with active acromegaly and normal glucose metabolism at baseline (pre-treatment)

After 6 months of therapy, the subjects with normal glucose profile at diagnosis did not significantly change the fasting glycaemia (89.71 mg/ dL vs. 93 mg/ dL, p=0.65) but the 2-h PG (OGTT) was statistically significantly lower than baseline (82.35 mg/ dL vs. 93 mg/ dL, p=0.005) according to the lowering of the GH and IGF1 levels, but only one subject had a complete control of acromegaly (by normal IGF1 and a GH level less than 1 ng/ mL in OGTT) (**Fig. 1**).

**Fig. 2:**
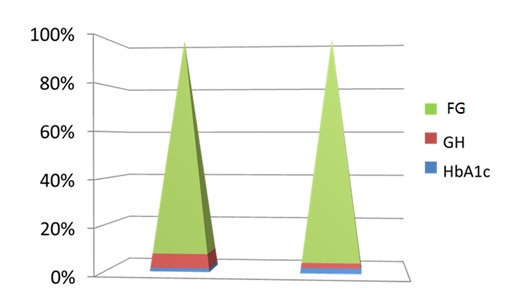
Fasting Glucose (FG, in mg/ dL); GH (Growth Hormone) levels (ng/ mL) and HbA1c (glycated haemoglobin, in %) after 6 months of octreotide LAR (a dose of 20 mg at every 28 days) in patients with active acromegaly and diabetes mellitus at baseline

The patients with DM had no statistically significant changes in A1c although GH considerably improved from a 24-h mean value of 33.12 ng/ mL to 6.92 ng/ mL (p<0.005) (**Fig. 2**). 

After 12 months of therapy, the patients with normal glycaemia pre-treatment profile had no statistically significant changes in FG and 2-h BG (in OGTT) despite the relevant lowering of GH: 3,3 ng/ mL vs. 6,16 ng/ mL, (p=0.003) and IGF1: 332 ng/ mL vs. 713 ng/ mL (p=0.001) (**Fig. 3a**,**b**). 

**Fig. 3a:**
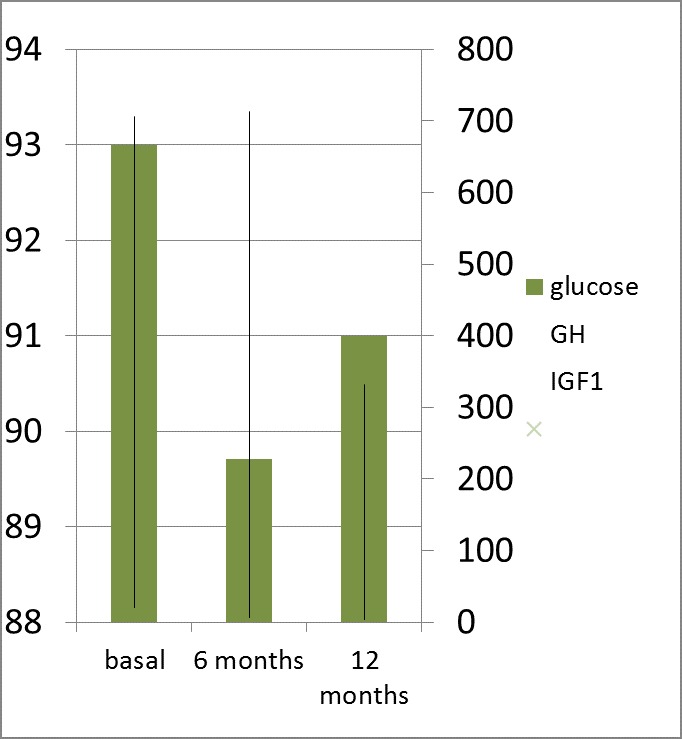
Fasting Glucose (Glucose; mg/ dL); GH (ng/ mL) and IGF1 (ng/ mL) levels after 12 months of octreotide LAR (a dose of 20 mg at every 28 days for 6 months followed by 6 months of 30 mg at every 28 days) in patients with active acromegaly and normal glucose metabolism at baseline (pre-treatment)

**Fig. 3b:**
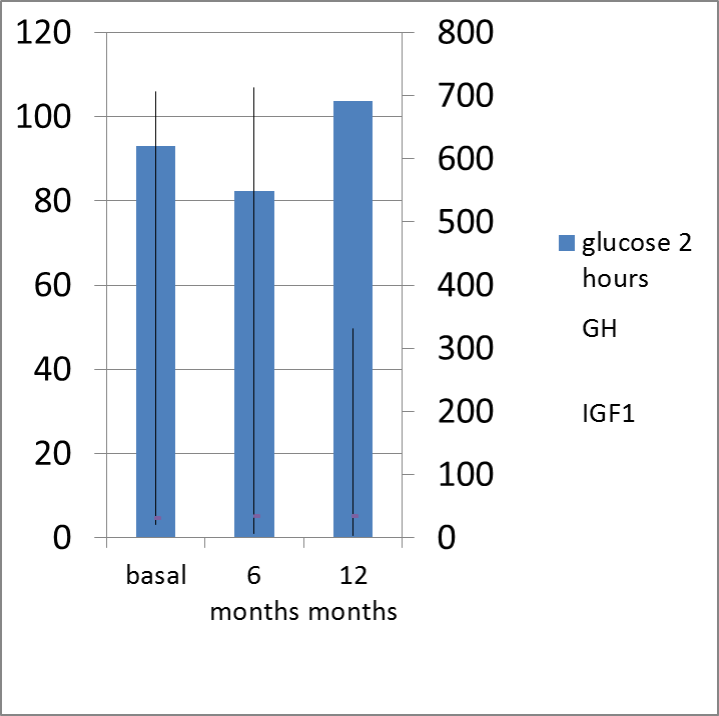
2-hour Plasma Glucose (Glucose 2 hours; mg/ dL) in 75 g OGTT; GH (ng/ mL) and IGF1 (ng/ mL) levels after 12 months of octreotide LAR (a dose of 20 mg at every 28 days for 6 months followed by 6 months of 30 mg at every 28 days) in patients with active acromegaly and normal glucose metabolism at baseline (pre-treatment)

**Fig. 4:**
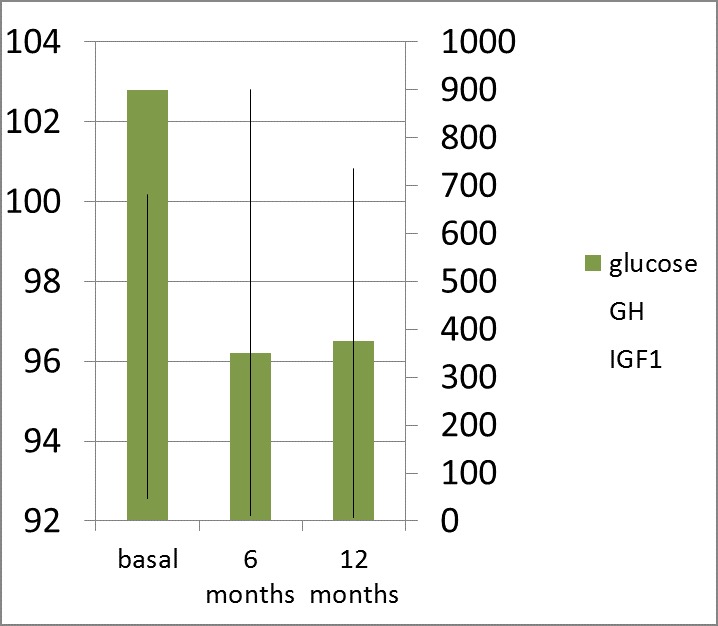
Fasting Glucose (Glucose; mg/ dL); GH (ng/ mL) and IGF1 (ng/ mL) levels after 12 months of octreotide LAR (a dose of 20 mg at every 28 days for 6 months followed by 6 months of 30 mg at every 28 days) in patients with active acromegaly and pre-diabetes (IGF+IGT) at baseline (pre-treatment)

Acromegalic patients with pre-treatment glucose metabolism considered as IGT or IFG had a statistically significantly reduction of FG after 6, respective 12 months according to GH and IGF1 lowering (**Fig. 4**). 

**Fig. 5:**
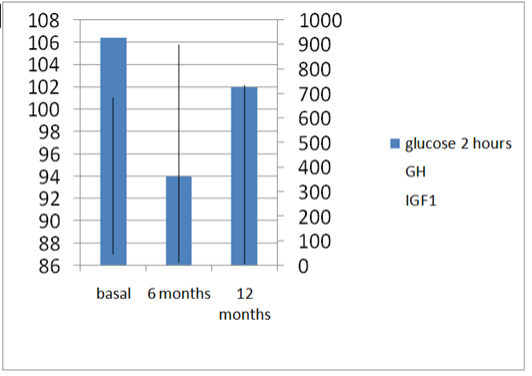
2-hour Plasma Glucose (Glucose 2 hours; mg/ dL) in 75 g OGTT; GH (ng/ mL) and IGF1 (ng/ mL) levels after 12 months of octreotide LAR (a dose of 20 mg at every 28 days for 6 months followed by 6 months of 30 mg at every 28 days) in patients with active acromegaly and pre-diabetes (IGF+IGT) at baseline (pre-treatment) (after 1 year: p<0.07)

The 2-h BG in OGTT after an initially (within first 6 months) decreasing later (during the next 6 months) increased back to the pre-treatment levels so no significant differences were seen after 1 year (p<0.07) (**Fig. 5**).

After 12 months both basal and 2-hours OGTT glucose levels were similar to baseline despite significant lower levels of GH (3.3 vs. 6.61 ng/ mL, p=0.003) and IGF1 (332 vs. 713 ng/ mL, p=0.001).

## Discussions

The glucose metabolism profile seems to be more related to the patients’ age than to the GH and IGF control [**9**]. In this study, DM had a greater incidence in subjects older than 61 years corresponding to similar values of GH and IGF1 from different age groups. Pre-treatment values of GH and IGF1 were not different if the patients had DM, IFG, IGT or normal glucose profile at baseline. While literature data pointed a good outcome of glucose metabolism after pituitary surgery, the somatostatin analogues had a variant effect. Initially, the insulin suppression caused glucose increasing despite GH suppression and later higher or lower glycaemia levels could be seen, regardless [**13**,**14**]. In acromegalic with DM, the fasting glucose and glycated haemoglobin A1c did not differ after 6 months of octreotide although the mean GH values were half as initial. IGF1 did not differ between either baseline or follow-up. Studies showed a previous hyperinsulinism mechanism in diabetic subjects with acromegaly characterised by normal fasting glucose with rapid and persistent insulin response in OGTT, which became normal again later than seen in normal conditions or IGT/ IFG [**15**-**17**]. This stage is reversible if adequate acromegaly therapy is introduced but later it will progress to a maximal beta pancreatic cells fasting response without further insulin enhance which is considered irreversible despite treatment [**18**-**20**]. This previous high insulin status would be able to explain the partial glucose answer to octreotide therapy in diabetic subjects although somatostatin analogues increase the insulin sensitivity by blocking the hepatic production of glucose [**18**-**20**]. These results are similar to those in literature according to which the glucose profile is mainly dependent on the baseline glycaemia status and the GH changes under octreotide. Moreover, we did not find higher glucose levels under therapy by beta cells inhibition caused by somatostatin analogue as some studies previously reported [**21**,**22**]. As limits of our study, we should mention the fact that we did not measure the insulin resistance indexes. 

## Conclusions

The therapy with octreotide in subjects with acromegaly improves the glucose profile if normal glycaemia is found at baseline according to GH and IGF1 lowering and, if the patients already have diabetes mellitus, only partial glucose control is found, as GH and IGF1 are inadequately suppressed. 

**Conflict of interest**

None.

**Acknowledgement**

We thank the entire medical team involved in this study.
